# Living with hepatitis C, multiple issues to face

**DOI:** 10.1186/1742-4690-9-S1-P52

**Published:** 2012-05-25

**Authors:** Mathilde Coudray, Elisabete de Carvalho

**Affiliations:** 1 Sida Info Service, Montpellier, France

## Aims

Since 2010, viral hepatitis has been declared a public health priority, although it remains little known to the general population. In France, 220.000 people are positive to the hepatitis C virus (HCV) and live with its chronic form, but limited data exists. This study was undertaken to assess the current face of the epidemic in France, and to identify the profiles of people living with HCV and their specific challenges.

## Methods

The survey consisted of a questionnaire accessible from November 2010 to February 2011 on the Hépatites Info Service website, a French hepatitis helpline. Callers meeting the criteria could also complete the questionnaire with a professional counselor on the toll-free line. After 3 months, 165 questionnaires had been collected.

## Results

Participants’ backgrounds are diverse with equal female to male ratio, and an average age of 50. Women are older and more likely to have been infected by blood transfusion prior to 1991 or by medical procedure with early stage diagnosis. Men, however, frequently presume to have been infected through intravenous drug use with late stage diagnosis. For all participants, HCV treatment presents a major challenge. Almost all (96%) undergoing or having concluded treatment emphasized difficult side effects: 26% interrupted treatment early and 69% took sick leave. Inadequate health insurance, solitude or addiction problems and coping with the social stigma of HCV infection multiply general difficulties.

## Conclusion

Living with HCV has major and multiple physiological and psychological consequences, in part treatment-associated. Economic and social precarity and solitude amid a context of discrimination combine to increase the issues confronting HCV positive people. The study has highlighted the diversity of profiles and problems which need to be integrated into a comprehensive care approach.

